# Controlling for unmeasured confounding and spatial misalignment in long‐term air pollution and health studies

**DOI:** 10.1002/env.2348

**Published:** 2015-07-26

**Authors:** Duncan Lee, Christophe Sarran

**Affiliations:** ^1^ School of Mathematics and StatisticsUniversity of GlasgowGlasgowU.K.; ^2^ UK Met OfficeExeterU.K.

**Keywords:** air pollution and health, spatial confounding, spatial misalignment

## Abstract

The health impact of long‐term exposure to air pollution is now routinely estimated using spatial ecological studies, owing to the recent widespread availability of spatial referenced pollution and disease data. However, this areal unit study design presents a number of statistical challenges, which if ignored have the potential to bias the estimated pollution–health relationship. One such challenge is how to control for the spatial autocorrelation present in the data after accounting for the known covariates, which is caused by unmeasured confounding. A second challenge is how to adjust the functional form of the model to account for the spatial misalignment between the pollution and disease data, which causes within‐area variation in the pollution data. These challenges have largely been ignored in existing long‐term spatial air pollution and health studies, so here we propose a novel Bayesian hierarchical model that addresses both challenges and provide software to allow others to apply our model to their own data. The effectiveness of the proposed model is compared by simulation against a number of state‐of‐the‐art alternatives proposed in the literature and is then used to estimate the impact of nitrogen dioxide and particulate matter concentrations on respiratory hospital admissions in a new epidemiological study in England in 2010 at the local authority level. © 2015 The Authors. Environmetrics published by John Wiley & Sons Ltd.

## 1 Introduction

The health effects of air pollution came to prominence in the mid‐1900s, as a result of high‐pollution episodes in the Meuse Valley in Belgium; Donora, Pennsylvania; and London, England. Air pollution concentrations are now greatly reduced in much of the world, but in April 2014, the World Health Organisation still estimated that outdoor air pollution was responsible for the deaths of 3.7 million people under the age of 60 in 2012. The impact of long‐term exposure to air pollution is typically estimated using cohort studies (Cesaroni *et al*., 2014), but the follow‐up period required for the cohort makes them time‐consuming and expensive to implement. Therefore, spatial ecological study designs are now being used, and examples include the works of Elliott *et al.* (2007) and Lee *et al.* (2009). These studies are inexpensive and quick to implement, owing to the now routine availability of the required data. Thus, while they cannot provide individual‐level evidence on cause and effect, they independently corroborate the body of evidence provided by cohort studies.

Spatial ecological studies utilise geographical contrasts in air pollution concentrations and population‐level disease risks over a set of non‐overlapping areal units, and the analysis of these data typically uses Poisson log‐linear models. The spatial pattern in disease risk is explained by known covariates and a set of spatially autocorrelated random effects, the former including air pollution concentrations and measures of socio‐economic deprivation and demography. The random effects account for any spatial autocorrelation remaining in the disease data after the covariate effects have been accounted for, which could be caused by unmeasured confounding, neighbourhood effects (where subjects' behaviour is influenced by that of neighbouring subjects) and grouping effects (where subjects choose to be close to similar subjects). This is achieved by modelling the random effects with a conditional autoregressive (CAR, Besag *et al.*) prior distribution, as part of a hierarchical Bayesian model. A relatively small number of these studies have been published to date (e.g. Jerrett *et al.*, 2005; Maheswaran *et al.*, 2005; Elliott *et al.*; 2007; Janes *et al.*, 2007; Lee *et al.*; 2009, Greven *et al.*; 2011; Lawson *et al.*, 2012), and the majority suffer from potential statistical limitations that could bias the estimated health risks.

The first limitation is the spatially smoothed random effects, which have been shown by Clayton *et al.* (1993) and Paciorek (2010) to be potentially collinear to spatially smooth covariates such as air pollution. Paciorek (2010) shows this potential collinearity depends on the closeness of the scales of spatial variation in air pollution and the random effects and can lead to poor estimation of the air pollution effect. Additionally, the disease data are unlikely to be globally spatially smooth, so the spatial pattern in the residuals after accounting for the known covariates is also unlikely to be globally smooth. Instead, the residuals are likely to exhibit localised autocorrelation, which is present between some pairs of adjacent areal units while other adjacent pairs exhibit very different residual values. Thus, traditional CAR models are insufficiently flexible to capture this localised spatial autocorrelation. The second limitation with existing models results from the pollution and disease data being spatially misaligned, as modelled pollution concentrations are available on a regular grid while the disease counts relate to irregularly shaped administrative units. Thus, the modelled pollution concentrations are at a finer spatial scale than the disease counts, resulting in variation in the pollution concentrations within an areal unit. This within‐area variation in concentrations has been ignored by the majority of existing studies (except for that of Haining *et al*., 2010) that compute the average concentration in each areal unit, which could give rise to ecological bias (Wakefield and Shaddick, 2006).

This paper presents a novel overarching Bayesian model with freely available software (the *CARBayes* package for the statistical software R, R Core Team, [Link]) for spatial air pollution and health studies, which is the first of its type to simultaneously address the dual problems outlined earlier. Secondly, it presents the first comparative assessment of a range of state‐of‐the‐art models used in the literature and quantifies the nature of any bias in their estimated pollution–health relationships. The statistical modelling issues highlighted earlier are motivated by a new study of air pollution and respiratory ill health in England in 2010, which is summarised in Section 2. Section 3 describes the statistical model proposed in this paper, while Section 4 quantifies the impact of inappropriate statistical modelling on the estimated air pollution effect via simulation. The results of the England study are presented in Section 5, while Section 6 presents a concluding discussion.

## 2 Description of the Study

**Figure 1:**
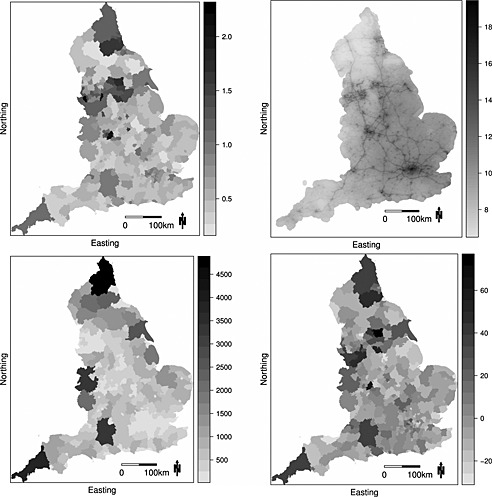
The top left panel displays the standardised morbidity ratio for hospital admissions due to respiratory disease in 2010, while the top right panel presents the modelled annual mean concentrations of particulate matter less than 2.5μm at a 1‐km^2^ resolution. The bottom left panel displays the number of modelled pollution concentrations in each local and unitary authority, while the bottom right panel displays the residuals from fitting a simple Poisson generalised linear model to the data

The study region is mainland England in 2010, and records of emergency hospital admissions due to respiratory disease were obtained from the Health and Social Care Information Centre and analysed by the UK Met Office. The resulting disease data are aggregated counts of the numbers of hospital admissions for each of the *n* = 323 local and unitary authorities (LUAs) in England, and the expected numbers of admissions based on national age‐specific and sex‐specific disease rates were also computed to adjust for differing population sizes and demographies across the LUAs. The simplest measure of disease risk is the standardised morbidity ratio (SMR), which is the ratio of the observed to the expected numbers of disease cases and is mapped in Figure 1 and summarised in Table 1. The figure shows the highest‐risk areas are cities in the north such as Liverpool and Manchester, while the lowest‐risk areas are typically rural such as West Somerset in the far south‐west of the country. The SMR map exhibits localised spatial smoothness, with some pairs of neighbouring LUAs having similar risks while other adjacent pairs are very different.

**Table 1:** Summary of the distribution of hospital admissions (as a standardised morbidity ratio), covariate and pollution data over the *n* = 323 local and unitary authorities in England

Variable	Distribution				
	0%	25%	50%	75%	100%
Standardised morbidity ratio	0.17	0.45	0.57	0.83	2.31
Log house price (£)	11.12	11.90	12.14	12.34	13.53
Jobs Seekers Allowance (%)	5.77	9.99	13.29	17.15	27.57
Mean					
NO_2_(μg m^−3^)	4.71	11.84	15.97	20.75	47.04
PM_2.5_(μg m^−3^)	6.87	9.76	10.76	11.64	16.74
PM_10_(μg m^−3^)	9.67	14.41	15.97	17.20	23.28
Coefficients of variation					
NO_2_	0.06	0.16	0.22	0.27	0.48
PM_2.5_	0.02	0.05	0.06	0.08	0.15
PM_10_	0.03	0.06	0.07	0.08	0.19

The pollution summaries relate to the distribution of means and coefficients of variation of the 1‐km concentrations within each local and unitary authority.

The network of air pollution monitors is sparse relative to the 323 LUAs, so modelled yearly average concentrations on a 1‐km^2^ grid available from http://uk-air.defra.gov.uk/data/pcm-data are used to characterise exposure. We consider concentrations of nitrogen dioxide (NO_2_) and particulate matter, the latter including both particles less than 2.5μm (PM_2.5_) and 10μm (PM_10_). The top right panel of Figure 1 displays the PM_2.5_ concentrations across England, from which the major cities and motorway network can be clearly seen. These modelled concentrations are spatially misaligned to the disease data, and the bottom left panel of Figure 1 shows that on average there are 215 pollution concentrations within a LUA, which ranges between 11 and 4889. The mean concentrations and coefficients of variation within each LUA are displayed in Table 1 for each pollutant, which shows that NO_2_ has the greatest relative levels of within‐LUA variation. The mean and variance of each pollutant within a LUA have positive and linear relationships, with correlation coefficients of 0.61 (NO_2_), 0.48 (PM_2.5_) and 0.16 (PM_10_).

Socio‐economic deprivation (poverty) has a major impact on disease risk (Mackenbach *et al.* 1997), as poorer populations typically exhibit greater tendencies for risk‐inducing behaviours, such as smoking or poor diet, than more affluent populations. However, data on these quantities are not available, so two proxy measures of poverty are used. The first is the percentage of the population in each LUA that are in receipt of Job Seekers Allowance, which Table 1 shows ranges between 5.77% and 27.57%. The second is the average property price in each LUA, which ranges between £67507 and £751630. A natural log transformation is used because exploratory analyses showed it exhibited a stronger relationship with disease risk. These covariates were included in a Poisson generalised linear model along with PM_2.5_ concentrations, and the residuals are displayed in the bottom right panel of Figure 1. They exhibit strong spatial autocorrelation, with a Moran I statistic of 0.282 (*p*‐value 0.00001). However, the autocorrelation is visually localised, with some pairs of neighbouring regions having very different residual values.

## 3 Modelling

This section outlines our proposed Bayesian hierarchical model, which is the first to simultaneously account for localised residual spatial autocorrelation and spatial misalignment between the pollution and disease data. The model is implemented in the R software environment, via the freely available *CARBayes* package.

### 3.1 Level 1—likelihood model

The vectors of observed and expected numbers of disease cases are denoted by **Y** = (*Y*_1_,…,*Y*_*n*_) and **E** = (*E*_1_,…,*E*_*n*_), respectively, where the latter is computed as $E_{k}=\underset{r}{\sum }N_{\mathrm{kr}}\gamma_{r}$ for area *k*. Here, *N*_*kr*_ is the size of the population in the *r*th age and sex stratum in areal unit *k*, while *γ*_*r*_ is the national strata‐specific disease rate. Let **w**={**w**_1_,…,**w**_*n*_} denote the air pollution data, where $w_{k}=(w_{k1},\ldots ,w_{kq_{k}})$ is the vector of *q*_*k*_ values within the *k*th area unit. These data are used to estimate **W** = (*W*_1_,…,*W*_*n*_), where *W*_*k*_ is a random variable representing the distribution of mean air pollution concentrations at different spatial locations within the *k*th areal unit. Finally, $X={\left(x_{1}^{T},\ldots ,x_{n}^{T}\right)}^{T}$ is a matrix of *p* known covariates, where $x_{k}^{T}=(1,x_{k2},\ldots ,x_{\mathrm{kp}})$ are the values for area *k*. We propose modelling **Y** with a Poisson log‐linear model of the form

(1) $$\begin{array}{cc} Y_{k}|E_{k},R_{k} & ∼\text{Poisson}(E_{k}R_{k})\;\text{for}\;\;k=1,\ldots ,n\\ R_{k} & =\exp\left(\overset{T}{\underset{k}{x}}\beta +\phi_{k}\right)g(W_{k};w_{k},\alpha ) \end{array}$$

Here, *R*_*k*_ is the risk of disease in area *k* relative to *E*_*k*_, and *R*_*k*_=1.2 corresponds to a 20% increased risk of disease. The covariate regression parameters ***β*** are assigned a multivariate Gaussian prior with weakly informative hyperparameters (***μ***_***β***_,Σ_***β***_). Section 3.2 describes our proposed localised spatial autocorrelation model for the random effects ***ϕ*** = (*ϕ*_1_,…,*ϕ*_*n*_), while Section 3.3 presents our modelling mechanism for *g*(*W*_*k*_;**w**_*k*_,*α*) that relates the air pollution concentrations **w**_*k*_ to the disease count *Y*_*k*_ while accounting for the spatial misalignment of these data.

### 3.2 Level 2—spatial autocorrelation model

Figure 1 shows the localised nature of the residual spatial autocorrelation in the disease data, and hence, we allow adjacent random effects (*ϕ*_*k*_,*ϕ*_*i*_) to be spatially autocorrelated or exhibit very different values, with the choice being determined by the data. We achieve this by partitioning *ϕ*_*k*_ into a globally smooth component *θ*_*k*_ and a piecewise constant intercept component $\lambda_{Z_{k}}$, which means that (*ϕ*_*k*_,*ϕ*_*i*_) will be similar if they have the same intercept (same $\lambda_{Z_{k}}$ value) but very different if they have different intercepts. The piecewise constant intercept surface has at most *G* distinct values ***λ*** = (*λ*_1_,…,*λ*_*G*_), and the model we propose is given by

(2) $$\begin{array}{cc} \phi_{k} & =\lambda_{Z_{k}}+\theta_{k}\;\text{for}\;\;k=1,\ldots ,n\\ \lambda_{i} & ∼\text{Uniform}(\lambda_{i-1},\lambda_{i+1})\;\text{for}\;\;i=1,\ldots ,G\\ f(Z_{k}) & =\frac{\exp(-\delta {\left(Z_{k}-G^{*}\right)}^{2})}{\overset{G}{\underset{r=1}{\sum }}\exp(-\delta {\left(r-G^{*}\right)}^{2})}\\ \delta & ∼\text{Uniform}(0,M=100)\\ \theta_{k}|\theta_{-k},\tau^{2},\rho ,W & ∼N\left(\frac{\rho \overset{n}{\underset{i=1}{\sum }}w_{\mathrm{ki}}\theta_{i}}{\rho \overset{n}{\underset{k=1}{\sum }}w_{\mathrm{ki}}+1-\rho },\;\frac{\tau^{2}}{\rho \overset{n}{\underset{k=1}{\sum }}w_{\mathrm{ki}}+1-\rho }\right)\\ \tau^{2} & ∼\text{Inverse‐gamma}(a=0.001,b=0.001)\\ \rho & ∼\text{Uniform}(0,1) \end{array}$$

The intercepts are ordered as *λ*_1_<*λ*_2_<⋯<*λ*_*G*_ to mitigate against label switching (with *λ*_0_=−*∞* and *λ*_*G* + 1_=*∞*), and area *k* is allocated to one of the *G* intercepts by *Z*_*k*_∈{1,…,*G*}. The maximum number of intercepts *G* is fixed in the model, but a shrinkage prior is assigned to each *Z*_*k*_ to penalise it towards the middle intercept term. This prior has the penalty term *δ*(*Z*_*k*_−G*)^2^, where G*=(*G* + 1)/2. If *G* is odd, then the prior shrinks each data point towards a single intercept term *λ*_G*_, while if *G* is even, it shrinks equally to (*λ*_G*−0.5_,*λ*_G*+0.5_). The latter will likely result in two different intercept terms being used to represent the data even if the residual structure is spatially smooth; thus, we recommend setting *G* to be an odd number. The size of *G* need only be small, as the intercept surface is designed to allow neighbouring areas to have very different random effects, and areas on different sides of the study region can have the same intercept value. Smooth spatial variation in the residual surface is modelled by ***θ*** = (*θ*_1_,…,*θ*_*n*_), and thus, ***λ*** models discrepancies from this smooth structure. We recommend setting *G* to be a small odd number such as 3 or 5 and test the sensitivity of this modelling assumption by simulation. A weakly informative uniform prior is specified for the penalty parameter *δ*, so that the data play the dominant role in estimating its value.

The spatially smooth variation in ***ϕ*** is represented by ***θ***, which is modelled by the CAR prior proposed by Leroux *et al.* (1999). Spatial autocorrelation is induced into these random effects via a binary *n* × *n* neighbourhood matrix **W**, where *w*_*ki*_=1 if areal units (*k*,*i*) share a common border and *w*_*ki*_=0 otherwise. The CAR prior given in (2) is defined by its full conditional distribution *f*(*θ*_*k*_|***θ***_−*k*_) for *k* = 1,…,*n*, where ***θ***_−*k*_=(*θ*_1_,…,*θ*_*k* − 1_,*θ*_*k* + 1_,…,*θ*_*n*_). It is a special case of a Gaussian Markov random field and can also be written as ***θ*** ∼ N(**0**,*τ* ^2^ **Q**(**W**,*ρ*)^−1^), where the precision matrix is given by **Q**(**W**,*ρ*) = *ρ*[diag(**W** **1**) − **W**] + (1 − *ρ*)**I** with (**1**,**I**) being a vector of ones and an identity matrix, respectively. This model induces a single level of spatial smoothness into ***θ***, with *ρ* = 1 corresponding to the intrinsic CAR prior for strong spatial smoothing proposed by Besag *et al.* (1991), while *ρ* = 0, corresponds to independent random effects. This can be better seen from the partial autocorrelation between (*θ*_*k*_,*θ*_*i*_) implied by this prior, which is given by

(3) $$\text{corr}\theta_{k},\theta_{i}|\theta_{-\mathrm{ki}}=\frac{\rho w_{\mathrm{ki}}}{\sqrt{\left(\rho \overset{n}{\underset{j=1}{\sum }}w_{\mathrm{kj}}+1-\rho \right)\left(\rho \overset{n}{\underset{l=1}{\sum }}w_{\mathrm{il}}+1-\rho \right)}}$$

Here, for all pairs of adjacent areal units, (*k*,*i*),*w*_*ki*_=1, and hence, they are partially autocorrelated with the level of that autocorrelation controlled globally for all adjacent pairs by *ρ*.

### 3.3 Level 3—the pollution–health relationship *g*(*W*_*k*_;**w**_*k*_,*α*)

As previously discussed, the pollution concentrations are spatially misaligned with the disease data, and the *k*th LUA has *q*_*k*_ concentrations $w_{k}=(w_{k1},\ldots ,w_{kq_{k}})$. The standard model for relating air pollution to health in the existing literature has the functional form

(4) $$R_{k}=\exp\left(\overset{T}{\underset{k}{x}}\tilde{\beta }+{\tilde{\phi }}_{k}\right)\exp({\hat{\mu }}_{k}\tilde{\alpha })$$

so that $g(W_{k};w_{k},\tilde{\alpha })=\exp(\mu_{k}\tilde{\alpha })$. Here, $\mu_{k}=E[W_{k}]$ and is estimated by ${\hat{\mu }}_{k}=(1/q_{k})\overset{q_{k}}{\underset{i=1}{\sum }}w_{\mathrm{ki}}$, the mean of the observed data **w**_*k*_. However, Wakefield and Shaddick 2006) have shown it is inappropriate to average the pollution concentrations via ((4), as this ignores the spatial variation in the concentrations within an areal unit. To see this, let *Y*_*ki*_, for *i* = 1,…,*q*_*k*_, denote the unknown number of disease cases from the population living in grid square *i* of areal unit *k* that experienced pollution concentration *w*_*ki*_. Then an appropriate model for the unobserved *Y*_*ki*_ would be *Y*_*ki*_∼Poisson(*E*_*ki*_ *R*_*ki*_), where $E_{\mathrm{ki}}=\underset{r}{\sum }N_{\mathrm{kir}}\gamma_{r}$, the expected number of cases of disease in grid square *i*. Here, *N*_*kir*_ is the population size of strata *r* in grid square *i* in areal unit *k*, and clearly, $E_{k}=\overset{q_{k}}{\underset{i=1}{\sum }}E_{\mathrm{kir}}$. The risk model again follows a log‐linear form as *Y*_*ki*_ is a count and is given by $R_{\mathrm{ki}}=\exp(\overset{T}{\underset{k}{x}}\beta +\phi_{k}+w_{\mathrm{ki}}\alpha )$. Here, $x_{k}^{T}\beta +\phi_{k}$ are measured and unmeasured covariates that have the same effect on disease risk across all *q*_*k*_ sub‐populations within areal unit *k*. The observed disease data are $Y_{k}=\overset{q_{k}}{\underset{i=1}{\sum }}Y_{\mathrm{ki}}$, and assuming conditional independence between *Y*_*ki*_|*E*_*ki*_,**x**_*k*_,*w*_*ki*_ across grid squares *i* yields $Y_{k}∼\text{Poisson}(\overset{q_{k}}{\underset{i=1}{\sum }}E_{\mathrm{ki}}R_{\mathrm{ki}})$. Simplifying this expression yields the aggregate likelihood model

(5) $$\begin{array}{cc} Y_{k}|E_{k},R_{k} & ∼\text{Poisson}(E_{k}R_{k})\;\text{for}\;\;k=1,\ldots ,n\\ R_{k} & =\exp\left(\overset{T}{\underset{k}{x}}\beta +\phi_{k}\right)\overset{q_{k}}{\underset{i=1}{\sum }}\overset{*}{\underset{\mathrm{ki}}{E}}\exp(w_{\mathrm{ki}}\alpha ) \end{array}$$

where $E_{\mathrm{ki}}^{*}=E_{\mathrm{ki}}/E_{k}$ and $\overset{q_{k}}{\underset{i=1}{\sum }}E_{\mathrm{ki}}^{*}=1$. This means that $g(W_{k};w_{k},\alpha )=\overset{q_{k}}{\underset{i=1}{\sum }}E_{\mathrm{ki}}^{*}\exp(w_{\mathrm{ki}}\alpha )$ in the general model (1), which is the same as equation (3.3) in Wakefield and Shaddick 2006) except that they use the total population size in each grid square as weights in the preceding sum rather than the expected number of cases $E_{\mathrm{ki}}^{*}$. Thus, the aggregate model ((5) differs from the naïve ecological model (4) in that $\overset{q_{k}}{\underset{i=1}{\sum }}E_{\mathrm{ki}}^{*}\exp(w_{\mathrm{ki}}\alpha )\neq \exp({\hat{\mu }}_{k}\tilde{\alpha })$, as in the left‐hand side the averaging over pollution is performed on the exponentiated risk scale while on the right‐hand side it is performed on the raw pollution scale (which is then exponentiated). In this paper, we compare the bias from using (4) rather than (5) by simulation in the next section but show in Section 2 of the supporting information that the potential bias can in part be predicted under a few simplifying assumptions. Finally, we note that as the pollution data are modelled estimates, they are subject to measurement error, but only a single concentration is available at each grid square without a corresponding measure of uncertainty. Therefore, the measurement error likely to be present in these data cannot be quantified, for example using a measurement error model. We also note that a classical measurement error model that treats the *q*_*k*_ observations as error‐prone measurements of an true unknown concentration in areal unit *k* is not appropriate, because this assumes there is a single and constant pollution concentration across each areal unit.

### 3.4 Alternative approaches

A number of approaches to dealing with residual spatial autocorrelation have been proposed in the literature, and we provide a brief review here and compare a number of them by simulation in Section 4. The simplest ignores the correlation and sets *ϕ*_*k*_=0, while the most common approach uses a CAR model similar to that proposed by Leroux *et al.* 1999). Reich *et al.* (2006) and Hughes and Haran (2013) replace the random effects with a set of basis functions that are orthogonal to the covariates, with those used by the latter also being spatially smooth. We compare the approach of Hughes and Haran (2013) with that proposed here in the simulation study, and a detailed description of their model is given in Section 3 of the supporting information accompanying this paper. The model proposed by Hughes and Haran (2013) does not capture localised spatial autocorrelation, and Lu *et al.* (2007) and Lee and Mitchell (2013) have proposed extensions to CAR models that capture such localised autocorrelation. For adjacent areal units, their approach models *w*_*ki*_∈**W** as a binary random variable, and if *w*_*ki*_=1, the corresponding random effects are partially autocorrelated, while if *w*_*ki*_=0, they are conditionally independent (Equation ((3)). Lee and Mitchell (2013) propose an iterative algorithm for estimation of **W**, which is compared in the simulation study in Section 4 and described fully in the supporting information (Section 3). Finally, Lawson *et al.* (2012) propose a two‐stage approach to modelling autocorrelation, where they first model the data with only the known covariates. They then model the residuals from this initial model with a space–time mixture structure in the second stage, allowing them to estimate the unmeasured spatio‐temporal autocorrelation structure in the data. Finally, they treat this estimated autocorrelation structure as an offset in a model including the known covariates.

## 4 Simulation Study

This section presents two simulation studies, which respectively assess the impact that different types of residual spatial confounding and within‐area variation in the pollution concentrations have on health effects estimation.

### 4.1 Study 1—spatial confounding

#### 4.1.1 Data generation and study design

Simulated disease counts *Y*_*k*_ are generated from a Poisson model similar to (1) for the *n* = 323 LUAs comprising mainland England, and the expected counts *E*_*k*_ are generated from a uniform distribution on the range [70,130] to give a moderate disease prevalence in terms of the existing literature. The log‐risk surface is a linear combination of a spatially smooth covariate acting as air pollution and residual spatial autocorrelation. For this first study, the pollution covariate is assumed to have no within‐area variation and is generated from a Gaussian spatial processes with a mean of 20 and a spatially smooth variance matrix defined by the Matérn family of autocorrelation functions. For the latter, the smoothness parameter equals 1.5, and the range parameter equals 60. A linear relationship is assumed between air pollution and health, and the regression parameter corresponds to a 5% increase in disease risk for a 2‐μg m^−3^ increase in pollution concentrations, which is similar to that estimated in Section 5.

Twelve different scenarios are considered for the spatial confounding component ***ϕ***, which cover the range of scenarios likely to be seen in real data. Firstly, the magnitude of this confounding is altered by fixing its standard deviation (denoted SD_***ϕ***_) at either 0.1 or 0.01. Six different spatial structures are generated for ***ϕ*** under both values of SD_***ϕ***_, where scenarios A–C correspond to a global level of spatial smoothness while scenarios D–F are locally smooth with step changes in ***ϕ*** between some pairs of adjacent areas. Here, scenario A corresponds to independence in space, scenario B is spatially autocorrelated but less smooth than the pollution covariate, while in scenario C ***ϕ*** is as smooth as the pollution covariate. Scenarios D–F mirror these global patterns but additionally have step changes in ***ϕ*** to represent localised smoothness. Example realisations of these spatial surfaces are displayed in Section 4 of the supporting information accompanying this paper, together with complete details of the data‐generating mechanism. Scenarios A–C correspond to setting *G* = 1 in Equation (2), while Scenarios D–F correspond to *G* = 3. We apply five models to each simulated data set, which include the localised smoothing model (2) proposed here (denoted *Model‐Local*), as well as a simple overdispersed Poisson log‐linear model (denoted *Model‐GLM*), a spatially smooth CAR model (denoted *Model‐CAR*) and the models of Hughes and Haran (2013) (denoted *Model‐HH*) for orthogonal autocorrelation and that of Lee and Mitchell (2013) (denoted *Model‐LM*) for localised autocorrelation.

#### 4.1.2 Results

Five hundred data sets are generated under each scenario, and the percentage bias and percentage root mean square error (RMSE) of the estimated air pollution effects are computed as $\text{Bias}(\alpha )=100\times (\frac{1}{500}\overset{500}{\underset{i=1}{\sum }}({\hat{\alpha }}_{i}-\alpha ))/\alpha$ and $\text{RMSE}(\alpha )=100\times (\sqrt{\frac{1}{500}\overset{500}{\underset{i=1}{\sum }}{\left({\hat{\alpha }}_{i}-\alpha \right)}^{2}})/\alpha$, respectively, where ${\hat{\alpha }}_{i}$ is the estimate (posterior median) for the *i*th simulated data set. Also computed are the coverage probabilities of the 95% uncertainty intervals from each model, and all the results are displayed in Table 2. The results presented for Model‐Local relate to *G* = 5 so as not to equal the true values (*G* = 1 and *G* = 3) that generated the data, but a sensitivity analysis to this choice is presented in Section 5 of the supporting information, which shows the results are largely insensitive to this choice.

**Table 2:** Results of the first simulation study

Scenario	SD_***ϕ***_	Model				
		Model‐GLM	Model‐CAR	Model‐Local	Model‐HH	Model‐LM
Bias						
A	0.1	0.39	0.41	−0.34	0.40	0.40
	0.01	0.18	0.16	0.07	0.20	0.14
B	0.1	0.31	−0.25	−0.80	0.33	−0.36
	0.01	−0.11	−0.12	−0.18	−0.08	−0.12
C	0.1	−0.26	−0.27	0.95	−0.23	−0.22
	0.01	−0.02	−0.02	0.10	0.02	−0.04
D	0.1	1.38	−1.31	−0.11	1.04	0.83
	0.01	0.17	1.02	−0.11	0.02	1.06
E	0.1	−2.18	−0.63	0.81	−1.10	0.76
	0.01	0.56	0.71	0.31	0.59	1.80
F	0.1	−2.63	−0.19	0.93	−1.69	1.15
	0.01	−2.84	−1.23	−0.04	−3.00	1.00
Root mean square error
A	0.1	6.37	6.43	6.45	6.43	6.45
	0.01	4.48	4.51	4.46	4.52	4.51
B	0.1	17.71	15.23	15.28	17.64	15.31
	0.01	4.90	4.90	4.91	4.90	4.90
C	0.1	26.70	18.73	18.91	26.44	18.34
	0.01	4.78	4.75	4.74	4.79	4.74
D	0.1	53.49	45.42	7.60	47.23	16.13
	0.01	52.65	43.88	4.84	45.47	9.02
E	0.1	59.71	49.87	16.88	53.18	19.48
	0.01	52.53	42.37	4.70	44.93	7.94
F	0.1	60.71	50.32	22.64	54.20	20.51
	0.01	53.98	43.81	4.64	47.02	8.39
Coverage
A	0.1	94.8	96.6	94.8	84.8	96.4
	0.01	94.0	96.6	96.2	95.4	96.8
B	0.1	52.8	85.4	85.6	42.8	87.6
	0.01	93.4	95.8	95.6	93.6	96.6
C	0.1	35.0	77.4	77.0	25.8	79.0
	0.01	93.4	96.0	96.2	93.6	96.8
D	0.1	67.8	91.2	94.4	16.4	95.0
	0.01	70.0	92.2	94.6	17.6	95.0
E	0.1	62.6	89.0	76.2	10.6	84.8
	0.01	68.4	93.8	94.0	17.8	97.8
F	0.1	63.6	87.4	63.2	17.0	74.6
	0.01	67.2	91.4	95.6	15.8	96.6

The top panel displays the bias (as a percentage of the true value) for the pollution–health relationship estimated by each of the five models, and the middle panel displays the root mean square error (as a percentage of the true value), while the bottom panel displays the coverage probabilities (as a percentage) of the 95% uncertainty intervals.

The top panel in Table 2 shows negligible bias in almost all cases, with percentage biases being lower than 2% in all but four cases and at most 3% overall. Scenario A corresponds to no spatial confounding as ***ϕ*** is independent in space, and all models perform similarly with less than 7% RMSE and coverages close to 95%. The only exception to this is for Model‐HH when SD_***ϕ***_=0.1, whose coverage is less than 85%. This relatively poor coverage for Model‐HH is consistently observed for the other scenarios and is caused in part by its relatively poor point estimation (as measured by RMSE) compared with most of the other models. The other possible reason for its poor coverage is that it includes a much lower‐dimensional (hence more parsimonious) set of random effects compared with most of the other models, which may thus lead to less variation being propagated through the model. The poor point estimation of Model‐HH occurs because its random effects are orthogonal to the fixed effects, whereas in Scenarios B and C, the simulated data are generated so that the residual spatial autocorrelation is potentially collinear to the fixed effects. The random effects in Model‐HH are also globally smooth, and thus, it cannot capture the localised step changes present in Scenarios D–F.

Scenarios B and C correspond to increasing spatial confounding, and all models exhibit dramatic rises in RMSE and falls in coverage when SD_***ϕ***_=0.1. Model‐CAR, Model–Local and Model‐LM perform best in this regard and have similar results, in terms of both RMSE and coverage. Model‐HH, which is designed to overcome this spatial confounding, does not outperform the other models that are prone to suffer from this confounding, for the reasons discussed earlier. When the standard deviation of ***ϕ*** drops to 0.01, the results are similar to those under Scenario A, because the level of spatial confounding is very small and hardly affects fixed‐effects estimation.

Finally, under the localised spatial confounding Scenarios D–F, Model‐Local performs best followed by Model‐LM in terms of RMSE, having lower RMSEs than the remaining models by up to 10 times. This is because they are the only models that allow for a non‐constant level of spatial smoothing across the study region and thus are able to accurately represent the localised spatial autocorrelation. However, the price for this is that Model‐Local performs worse in terms of coverage than the globally smooth CAR model (Model‐CAR) in Scenarios E and F when SD_***ϕ***_=0.1. This is because the piecewise constant intercept term in Model‐Local accurately captures the step changes in the localised residual structure, leaving the random effects ***θ*** to represent the remaining smooth spatial structure. However, this remaining smooth structure is collinear to the fixed effect, resulting in reduced coverage as observed in Scenarios B and C. This does not occur for scenario D, where this remaining structure is independent in space and thus not collinear to the covariate. In contrast, this drop in coverage does not happen to the same extent for Model‐CAR, because its globally smooth random effects are trying to model the localised structure, which they are not designed to do. The result is a largely inflated random‐effects variance *τ* ^2^, as the amount of smoothing to the spatially smooth mean function in the CAR prior is reduced. This inflated level of variation causes a similar increase in variation in the posterior distribution of the fixed effect, leading to greater coverage. However, this increase in coverage comes at the cost of a large (up to 10 times) decrease in the accuracy of its point estimation.

### 4.2 Study 2—within‐area variation in pollution

#### 4.2.1 Data generation and study design

Simulated data are generated using the same approach as in the first study, except that the pollution concentrations vary within each LUA. The number of concentrations observed for each LUA is the same as in the real data, and we examine the impact that this variation has on the estimated health effects under a number of scenarios. We vary the size of the estimated health effects and the type of within‐LUA variation in pollution, as the discussion in Section 3 and the supporting information suggests that both will affect the results. The pollution–health relationships considered here have relative risks of 1.05 and 1.5 for a 2‐μg m^−3^ increase in concentrations, and the latter is chosen to be overly large. The standard deviation of pollution within each LUA is fixed at 1 or 10 and is also allowed to be independent or positively linearly related to the mean pollution level in an LUA. In all cases, the distribution of pollution concentrations within an LUA is assumed to be Gaussian. Finally, we also investigate the impact of changing the scaled expected counts $\left(E_{k1}^{*},\ldots ,E_{kq_{k}}^{*}\right)$ and first assume they are all equal, that is, $E_{\mathrm{ki}}^{*}=1/q_{k}$. We then relax this assumption and generate them from a uniform distribution on the unit interval (with appropriate rescaling). In all scenarios, we compare the aggregate model (5) proposed here with the naïve ecological risk model ((4), with a Poisson data likelihood) commonly used in these studies, and both are completed by (2).

#### 4.2.2 Results

Five hundred data sets are generated under each of the 16 scenarios described earlier, and the results presented in Table 3 include the percentage bias, percentage RMSE and coverage probabilities for the estimated pollution–health relationships. The top panel of the table displays results for constant $E_{\mathrm{ki}}^{*}=1/q_{k}$, while the bottom panel corresponds to them varying within an LUA. The table shows broadly similar bias, RMSE and coverage results under the two specifications of $\left(E_{k1}^{*},\ldots ,E_{kq_{k}}^{*}\right)$, suggesting that a non‐constant set of within‐LUA expected counts does not impact health effects estimation. The table also shows that, when the pollution–health effect has a similar size to that observed in the literature, neither the ecological model nor the aggregate model exhibits any systematic bias, regardless of the level of within‐area variation in the pollution concentrations. This is because the bias term 0.5*b* *α* ^2^ (based on the Gaussian and linearity assumptions, see the supporting information) in the naïve ecological model is small as the true value of *α* = 0.0244. The RMSE values and coverage probabilities for both models are similar, with the latter being close to their nominal 95% levels. These results thus suggest that while the ecological model is inappropriate mathematically, the small effect sizes seen in air pollution and health studies render its bias negligible in practice in this context. Conversely, for the larger effect size of a relative risk of 1.5 for a 2‐μg m^−3^ increase in pollution (*α* = 0.203), the ecological model shows large bias, large RMSE and low coverage if the within‐area variation in the pollution concentrations increases with the mean in a linear fashion. This result conforms to our theoretical expectations discussed in the supporting information, while the aggregate model does not suffer from these problems. Finally, as expected, if the within‐area variation in the pollution concentrations is independent of the mean pollution level, then the ecological model is once more unaffected.

**Table 3:** The table displays the bias, root mean square error (RMSE, both as a percentage of the true value) and the coverage probabilities for the pollution–health relationship estimated by the naïve ecological model (4) and the aggregate model (5), as both the true risk and the within‐area variation in pollution vary under different assumptions about $E_{\mathrm{ki}}^{*}$

Risk (*α*)		Pollution	Bias		RMSE		Coverage	
			Model (4)	Model (5)	Model (4)	Model (5)	Model (4)	Model (5)
Constant $E_{\mathrm{ki}}^{*}$							
1.05		SD = 1, independent	−0.31	−0.29	6.06	6.08	95.3	95.5
1.05		SD = 1, linear	−0.16	−0.22	5.85	5.83	96.2	96.4
1.05		SD = 10, independent	0.11	0.10	5.69	5.67	95.7	95.5
1.05		SD = 10, linear	0.45	−0.17	5.81	5.74	94.8	95.6
1.5		SD = 1, independent	−0.22	−0.34	14.06	13.92	95.4	94.4
1.5		SD = 1, linear	17.96	0.47	23.00	10.56	73.4	95.4
1.5		SD = 10, independent	−1.17	−0.64	7.87	5.35	92.6	94.3
1.5		SD = 10, linear	20.72	−0.09	23.57	2.37	44.6	95.9
Variable $E_{\mathrm{ki}}^{*}$							
1.05		SD = 1, independent	−0.07	−0.06	5.79	5.80	95.4	95.6
1.05		SD = 1, linear	0.40	0.33	5.80	5.78	94.8	94.4
1.05		SD = 10, independent	0.25	0.23	5.65	5.64	95.6	95.6
1.05		SD = 10, linear	0.91	0.26	5.71	5.54	95.0	95.0
1.5		SD = 1, independent	0.18	0.35	14.71	13.34	95.0	96.2
1.5		SD = 1, linear	17.21	−0.46	22.75	10.19	73.0	94.2
1.5		SD = 10, independent	−0.70	−0.44	8.61	4.47	91.4	96.0
1.5		SD = 10, linear	21.33	−0.02	24.98	2.04	46.6	97.0

## 5 Results from the England Study

### 5.1 Modelling

All models compared in the simulation studies were applied to the England data described in Section 2, which include the full aggregate model comprising (2) and (5) (*Model‐Local‐Agg*) proposed here (with *G* = 5) and the naïve ecological model comprising (2) and (4) with a Poisson data likelihood (Model‐Local, also with *G* = 5) that ignores within‐area variation in the pollution concentrations. The different models for residual spatial confounding are also applied to these data, which include a simple generalised linear model (Model‐GLM), a globally smooth CAR model (Model‐CAR, (1) and (2) with *ϕ*_*k*_=*θ*_*k*_) and the recent approaches of Hughes and Haran (2013) (Model‐HH) for orthogonal autocorrelation and Lee and Mitchell (2013) (Model‐LM) for localised autocorrelation.

The covariates included in each model are one of the three pollutants considered here (NO_2_, PM_2.5_, and PM_10_) and the proxy measures of socio‐economic deprivation, the latter including the percentage of the working age population in receipt of Job Seekers Allowance and the natural log of the median property price in each LUA. A single pollutant was included in each model because of the positive pairwise correlations between pollutants, which ranged between 0.79 and 0.94 at the LUA level. The expected numbers of respiratory hospital admissions were computed for the 7665 wards in England, which allows the pollution concentrations within each LUA to be weighted by $\left(E_{k1}^{*},\ldots ,E_{kq_{k}}^{*}\right)$ as described in Equation (5) for Model‐Local‐Agg. The remaining models use the mean concentration in each LUA as described in (4). Inference for all the Bayesian models (i.e. not Model‐GLM) was based on 100000 Markov chain Monte Carlo samples, which were generated from five parallel Markov chains, and convergence was visually checked by examining trace plots of sample parameters including the pollution–health relationship. The main study results are presented in the next subsection, while the following subsection presents some additional sensitivity analyses.

### 5.2 Main results

The estimated relationships between each pollutant and respiratory hospital admissions from each model are displayed in the top panel of Table 4 and are presented as relative risks for a realistic increase in pollution concentrations. These increases are 5μg m^−3^ for NO_2_ and 1μg m^−3^ for PM_2.5_ and PM_10_, because NO_2_ has larger average concentrations and larger variation across England. Overall, the table shows evidence of substantial relationships between air pollution and respiratory disease risk, as 17 of the 18 estimated relative risks have 95% uncertainty intervals that do not include the null risk of 1. The most definitive effects are observed for NO_2_ and PM_2.5_, while weaker effects are observed for PM_10_.

**Table 4:** Estimated relative risks and 95% uncertainty intervals (confidence intervals for Model‐GLM and credible intervals for the remaining models) for 5‐μg m^−3^ (NO_2_) and 1‐μg m^−3^ (PM_2.5_ and PM_10_) increases in pollution concentrations from the models considered in this paper

Model	Pollutant		
	NO_2_	PM_2.5_	PM_10_
Main results			
Model‐GLM	1.085 (1.052, 1.118)	1.032 (1.005, 1.060)	1.008 (0.989, 1.027)
Model‐CAR	1.094 (1.055, 1.133)	1.055 (1.022, 1.094)	1.037 (1.014, 1.062)
Model‐Local	1.089 (1.071, 1.104)	1.032 (1.017, 1.047)	1.013 (1.003, 1.023)
Model‐Local‐Agg	1.086 (1.072, 1.100)	1.035 (1.021, 1.054)	1.010 (1.001, 1.019)
Model‐HH	1.088 (1.086, 1.091)	1.046 (1.044, 1.047)	1.019 (1.017, 1.020)
Model‐LM	1.091 (1.077, 1.105)	1.047 (1.033, 1.060)	1.033 (1.023, 1.043)
Sensitivity analysis			
*G* = 3	1.084 (1.065, 1.105)	1.035 (1.021, 1.051)	1.008 (0.999, 1.017)
*a* = 0.1,*b* = 0.1	1.085 (1.071, 1.110)	1.035 (1.024, 1.047)	1.010 (1.002, 1.020)
*a* = 0.5,*b* = 0.0005	1.084 (1.068, 1.099)	1.034 (1.022, 1.046)	1.010 (1.002, 1.018)
No population weighting	1.089 (1.073, 1.105)	1.030 (1.017, 1.046)	1.010 (1.001, 1.026)

The table also shows clear evidence that the model used to allow for residual spatial autocorrelation can have a large impact on health effects estimation, although the relative risks for NO_2_ are relatively stable ranging between 8.5% and 9.4% increases in disease risk for a 5‐μg m^−3^ increase in concentrations. However, the results for PM_2.5_ and PM_10_ show large variation in the estimated effect sizes, ranging between 3.2% and 5.5% increases for PM_2.5_ and between 0.8*%* and 3.7% for PM_10_. The largest estimated effect sizes are obtained from Model‐CAR for each pollutant, while those from Model‐Local are always smaller. Of course, one is unable to say which estimate is ‘correct’, but the results from Scenarios D–F in the simulation study in Section 4 suggest that as the spatial autocorrelation after accounting for the measured covariates is localised for these data (Figure 1), then Model‐Local is likely to produce the most accurate effect estimates. Further supporting evidence comes in the form of overall model fit, as the deviance information criterion (Spiegelhalter *et al.*, 2002) values are 3607.9 (*p*.*d* = 311.0) and 3535.7 (*p*.*d* = 234.6) for Model‐CAR and Model‐Local, respectively, suggesting a better model fit for the latter. An interesting side note is that the effective number of parameters *p*.*d* is lower for Model‐Local compared with that for Model‐CAR, even though the former has additional intercept and allocation parameters (*λ*_*i*_,*Z*_*k*_). This is because the inclusion of the piecewise constant intercept terms in Model‐Local means the random effects ***θ*** are smoothed more than in Model‐CAR (posterior medians for the variance *τ* ^2^ are 0.0131 and 0.1809, respectively), as they do not have to account for the localised residual spatial structure. Finally, a comparison of Model‐Local and Model‐Local‐Agg shows that ignoring within‐area variation in the pollution concentrations has little effect on the results, which was suggested by the simulation study as the estimated effect sizes $\left(\hat{\alpha }\right)$ are relatively small.

### 5.3 Sensitivity analyses

We then undertook a sensitivity analysis to assess the impact of changing the model assumptions of Model‐Local‐Agg, and the results are displayed in the bottom panel of Table 4. This analysis included the following: (i) changing the maximum number of risk classes *G* from five to three; (ii) changing the hyperparameters (*a*,*b*) of the inverse‐gamma distribution for *τ* ^2^ from (*a* = 0.001,*b* = 0.001) to (*a* = 0.1,*b* = 0.1) and (*a* = 0.5,*b* = 0.0005); and (iii) removing the weighting by $\left(E_{k1}^{*},\ldots ,E_{kq_{k}}^{*}\right)$ in (5) to give equal weight to all pollution concentrations. The table shows that changing *G* and (*a*,*b*) had no impact on the results, as the estimated relative risks for all pollutants remained almost identical. Removing the population weighting also had little effect, although the results for NO_2_ and PM_2.5_ changed by around 0.5%.

## 6 Discussion

This paper is the first to propose an integrated modelling framework for estimating the long‐term effects of air pollution on human health, accounting for localised spatial autocorrelation in the disease data and the inherent spatial misalignment between the exposure and the response. The model proposed here is widely applicable to geographical association studies beyond the air pollution arena and is available for other researchers to use via the R package *CARBayes* available free from http://www.R-project.org/. This paper also provides an in‐depth simulation study into the impact of spatial autocorrelation and spatial misalignment on fixed‐effects estimation and presents a new study into the long‐term effects of air pollution on respiratory disease in England in 2010.

One of our main findings is that inappropriate control for residual (i.e. after the effects of known covariates have been removed) spatial autocorrelation in the disease data can result in incorrect fixed‐effects estimation. This problem encompasses both point estimation and uncertainty quantification, and the first simulation study presented here shows some interesting results. Firstly, if, as is the case with the respiratory admissions data presented here, the residual spatial autocorrelation is not globally smooth, then wrongly assuming it is leads to substantially poorer estimation of covariate effects (in terms of RMSE) compared with using a localised smoothing model. Differences in fixed‐effects estimates were also seen empirically in the real data in Section 5, although one is of course unable to say which estimate is ‘correct’ in this case. The other main finding from the simulation study is that, as expected, the greater the level of confounding between the fixed effects and the residual spatial structure, the poorer all models do in terms of fixed‐effects estimation. This poor performance again encompasses point estimation and uncertainty quantification. However, what is surprising is that the commonly used CAR model, which has been subject to recent criticisms by Reich *et al.* (2006) and others, does no worse than other more sophisticated models and in fact outperforms the orthogonal smoothing model proposed by Hughes and Haran (2013).

The other main finding of this paper is that for air pollution and health studies, where effect sizes are typically small, ignoring the within‐area variation in the exposure caused by the spatial misalignment of the data does not lead to systematic bias. This result is illustrated in the second simulation study presented in Section 4 and is corroborated by the real‐data results presented in Section 5. The magnitude of any such bias depends on a number of factors, including the effect size being estimated, the distributional shape of the within‐area variation in the exposure, and the relationship between the mean and higher‐order moments of the within‐area exposure distribution. However, although no bias was observed here, in general, within‐area variation in an exposure should not be averaged away by computing a mean, as bias can result if the aforementioned conditions are right as shown in the simulation study in Section 4.

The spatial air pollution and disease data used in this study are routinely available for multiple consecutive time periods, and in future work, we will extend the model proposed here to the spatio‐temporal domain. Furthermore, the modelled concentrations used here have complete spatial coverage but are modelled estimates rather than measured concentrations and as such are prone to biases and uncertainties. However, no information on these are available, and thus, we plan to develop a two‐stage modelling approach for these data, where the first stage provides better estimates of pollution by fusing the modelled concentrations with observed monitor data using techniques similar to that of Berrocal *et al.* (2009). This approach would thus allow for the measurement error in the pollution data to be correctly propagated into the disease model, which was not possible for the data used in this paper.

## Supporting information



Supporting info item
