# Supplementary material for: Controlling for unmeasured confounding and spatial misalignment in long‐term air pollution and health studies
Source: Environmetrics. 2015 Jul 26;26(7):477–87. doi: 10.1002/env.2348 (PMC4975605; doi:10.1002/env.2348)
Supplement: Supplementary file 1 — Supporting info item [file ENV-26-477-s001.pdf]

Environmetrics 00, 1–10

DOI: 10.1002/env.XXXX

Supplementary material to 'Controlling for unmeasured confounding and spatial misalignment in long-term air pollution and health studies'

Duncan Lee[a*] and Christophe Sarran[b]

1. INTRODUCTION

This supplementary material accompanies the main paper and contains the following content. Section 2 derives an analytic form for the ecological bias that arises under the naive model (4), while Section 3 describes two of the state-of-the-art models proposed in the literature to account for spatial autocorrelation. Section 4 gives examples of the spatial data generated in the simulation study. Finally, Section 5 presents an additional simulation study assessing the sensitivity of **Model-Local** to the choice of the number of intercept terms G.

2. DERIVATION OF ECOLOGICAL BIAS

From the main paper the naive ecological and aggregate models have the following risk specifications:

[a] *School of Mathematics and Statistics, University of Glasgow, Glasgow, UK*
[b] *UK Met Office, Exeter, UK.*
[*] *Correspondence to: Duncan Lee, School of Mathematics and Statistics, University of Glasgow, Glasgow, G12 8QW, UK. E-mail: Duncan.Lee@glasgow.ac.uk*

This paper has been submitted for consideration for publication in *Environmetrics*

$$
\begin{aligned}
\textbf{Ecological} \quad R_k &= \exp(\mathbf{x}_k^{\mathrm{T}}\tilde{\boldsymbol{\beta}} + \tilde{\phi}_k)\exp(\hat{\mu}_k\tilde{\alpha}), \\
\textbf{Agregate} \quad R_k &= \exp(\mathbf{x}_k^{\mathrm{T}}\boldsymbol{\beta} + \phi_k)\sum_{i=1}^{q_k} E_{ki}^{*}\exp(w_{ki}\alpha).
\end{aligned}
$$

Assuming that $E_{ki}^{*} = 1/q_k$ for all grid squares i in areal unit k, the pollution component of the aggregate risk model simplifies to $(1/q_k)\sum_{i=1}^{q_k}\exp(w_{ki}\alpha)$. This is the sample equivalent of $\mathbb{E}[\exp(W_k\alpha)]$, the moment generating function of the random variable W_k that characterises the distribution of average pollution levels spatially within areal unit k. If you further assume that $W_k \sim \mathrm{N}(\mu_k, \sigma_k^2)$, then the moment generating function is given by $\exp(\mu_k\alpha + 0.5\alpha^2\sigma_k^2)$. This means that the correct risk model has pollution component $\exp(\hat{\mu}_k\alpha + 0.5\alpha^2\hat{\sigma}_k^2)$ (with the sample mean and variance replacing the unknown theoretical values) rather than $\exp(\hat{\mu}_k\tilde{\alpha})$ from the naive ecological model. If the mean and variance $(\hat{\mu}_k, \hat{\sigma}_k^2)$ are independent then no bias occurs, but this is not the case if the variance depends on the mean. For example, if the variance of W_k increases with the mean in a linear fashion, as is approximately the case with the data presented in Section 2 of the main paper, then $\hat{\sigma}_k^2 = a + b\hat{\mu}_k$ where $b > 0$. Then comparing the multipliers of $\hat{\mu}_k$ in the ecological and aggregate models shows that $\tilde{\alpha} = \alpha + 0.5b\alpha^2$. Thus as the effect size α is small for air pollution and health studies, the bias should be small due to the α^2 in the bias term $0.5b\alpha^2$.

3. MODELS FOR SPATIAL AUTOCORRELATION

Here we describe the models proposed by Hughes and Haran (2013) and Lee and Mitchell (2013) for modelling spatial autocorrelation in ecological regression studies, as they are compared to the model proposed in this paper in the simulation study in the main paper.

3.1. Model by Hughes and Haran (2013)

The orthogonal smoothing model of Hughes and Haran (2013) replaces the random effects $\boldsymbol{\phi}$ in (1) in the main paper with a linear combination of basis functions that are orthogonal to the covariates. Denote the matrix of covariates in model (1) in the main paper for all n observations as $\tilde{\mathbf{X}} = (\hat{\boldsymbol{\mu}}, \mathbf{X})$, where $\hat{\boldsymbol{\mu}}$ is the vector of estimated pollution concentrations for all n areal units described in (4) in the main paper. The residual projection matrix from a normal linear model based on this extended covariate matrix is given by

$$\mathbf{P} \;=\; \mathbf{I}_n - \tilde{\mathbf{X}}(\tilde{\mathbf{X}}^{\mathrm{T}}\tilde{\mathbf{X}})^{-1}\tilde{\mathbf{X}}^{\mathrm{T}}.$$

The basis functions included in the disease model come from the set of eigenvectors of the matrix product **PWP**, where **W** is the binary neighbourhood matrix determining the spatial adjacency structure of the areal units. Thus this matrix product combines spatial information via **W** with covariate orthogonality via **P**. Hughes and Haran (2013) show that the eigenvectors of **PWP** correspond to all possible mutually distinct patterns of spatial clustering orthogonal to the covariates, and that the eigenvectors corresponding to the positive eigenvalues relate to positive spatial autocorrelation. Additionally, the magnitude of the jth eigenvalue λ_j also determines the relative importance of the spatial pattern in the jth eigenvector, so Hughes and Haran (2013) suggest choosing the first $q << n$ eigenvectors corresponding to positive and decreasing eigenvalues. Denote this $n \times q$ matrix of eigenvectors by $\mathbf{M}$, where $\mathbf{m}_k^{\mathrm{T}} = (m_{k1}, \ldots, m_{kq})$ is the kth row. Here q is a tuning parameter in the model, with large values leading to less dimension reduction. The model proposed by Hughes and Haran (2013) is given by

$$
\begin{aligned}
Y_k|E_k, R_k &\sim \text{Poisson}(E_k R_k) \quad \text{for } k = 1, \ldots, n, \qquad (1)\\
R_k &= \exp(\mathbf{x}_k^{\mathrm{T}}\boldsymbol{\beta} + \hat{\mu}_k\alpha + \mathbf{m}_k^{\mathrm{T}}\boldsymbol{\delta}),\\
\boldsymbol{\delta} &\sim \text{N}(\mathbf{0}, \tau^2\mathbf{Q}(\mathbf{W})_s^{-1}).
\end{aligned}
$$

Here $\boldsymbol{\delta}$ has a Gaussian prior with a precision matrix given by $\mathbf{Q}(\mathbf{W})_s = \mathbf{M}^{\mathrm{T}}\mathbf{Q}(\mathbf{W})\mathbf{M}$, where $\mathbf{Q}(\mathbf{W}) = \text{diag}(\mathbf{W}\mathbf{1}) - \mathbf{W}$ is the precision matrix for the intrinsic CAR prior.

3.2. Model by Lee and Mitchell (2013)

The model proposed by Lee and Mitchell (2013) has the general form

$$
\begin{aligned}
Y_k|E_k, R_k &\sim \text{Poisson}(E_k R_k) \quad \text{for } k = 1, \ldots, n, \qquad (2)\\
R_k &= \exp(\mathbf{x}_k^{\mathrm{T}}\boldsymbol{\beta} + \hat{\mu}_k\alpha + \phi_k),\\
\phi_k|\boldsymbol{\phi}_{-k}, \tau^2, \rho, \mathbf{W} &\sim \text{N}\left(\frac{\rho\sum_{i=1}^{n} w_{ki}\phi_i}{\rho\sum_{k=1}^{n} w_{ki} + 1 - \rho}, \frac{\tau^2}{\rho\sum_{k=1}^{n} w_{ki} + 1 - \rho}\right),\\
\tau^2 &\sim \text{Inverse-gamma}(a = 0.001, b = 0.001),\\
\rho &\sim \text{Uniform}(0, 1),
\end{aligned}
$$

which is fitted using Integrated Nested Laplace Approximations (INLA, Rue et al (2009)) rather than McMC simulation. The model is iteratively re-fitted using different but fixed neighbourhood matrices $\mathbf{W}$, until a convergence criterion is met. If $w_{kj} \in \mathbf{W}$ is estimated as one then (ϕ_k, ϕ_j) are smoothed towards each other in the modelling process (see equation (3) in the main paper), otherwise $w_{kj} = 0$ and the two random effects are conditionally independent and are not smoothed towards each other. Thus allowing the elements of $\mathbf{W}$ to

be estimated allows for localised spatial smoothing. The algorithm for jointly estimating the model parameters $\mathbf{\Theta} = (\boldsymbol{\beta}, \alpha, \boldsymbol{\phi}, \tau^2, \rho)$ and the neighbourhood matrix $\mathbf{W}$ is as follows.

Algorithm

1: Estimate a starting posterior distribution for model (2) using INLA, which is denoted by $f(\mathbf{\Theta}^{(0)}|\mathbf{Y}, \mathbf{W}^{(0)})$. For this initial model we assume the random effects are independent, which is achieved by restricting model (2) by fixing $\rho = 0$.

2: Iterate the following two steps for $i = 1, 2, \ldots, i^*$, until one of the two termination conditions for the neighbourhood matrix $\mathbf{W}$, outlined in step 3, are met.

a: Estimate $\mathbf{W}^{(i)}$ deterministically from the current posterior distribution $f(\mathbf{\Theta}^{(i-1)}|\mathbf{Y}, \mathbf{W}^{(i-1)})$. Set $w_{kj}^{(i)} = 1$ if the marginal 95% posterior credible intervals for $(\phi_k^{(i-1)}, \phi_j^{(i-1)})$ overlap and areas (k, j) share a common border. Otherwise, set $w_{kj}^{(i)} = 0$.

b: Estimate the posterior distribution $f(\mathbf{\Theta}^{(i)}|\mathbf{Y}, \mathbf{W}^{(i)})$ of model (2) using INLA.

3: After i^* iterations one of the following two termination conditions will apply.

Case 1 - The sequence of $\mathbf{W}$ estimates is such that $\mathbf{W}^{(i^*)} = \mathbf{W}^{(i^*+1)}$, which is the estimated neighbourhood matrix $\hat{\mathbf{W}}$.

Case 2 - The sequence of $\mathbf{W}$ estimates forms a cycle of k different states $(\mathbf{W}^{(i^*)}, \mathbf{W}^{(i^*+1)}, \ldots, \mathbf{W}^{(i^*+k-1)}, \mathbf{W}^{(i^*+k)})$, where $\mathbf{W}^{(i^*)} = \mathbf{W}^{(i^*+k)}$. In this case the estimated neighbourhood matrix $\hat{\mathbf{W}}$ is the value from the cycle of k states that has the minimal level of residual spatial autocorrelation, as measured by the absolute value of Moran's I statistic, a measure of spatial autocorrelation.

When one of the termination conditions has been met $\hat{\mathbf{W}}$ is the estimated spatial structure of the random effects, and $\boldsymbol{\Theta}$ is summarised by the posterior distribution $f(\boldsymbol{\Theta}|\mathbf{Y}, \hat{\mathbf{W}})$.

4. SIMULATION STUDY- DATA GENERATION

Simulated disease count data Y_k are generated for the $n = 323$ local and unitary authorities comprising mainland England, using the following model:

$$\begin{aligned} Y_k|E_k, R_k &\sim \text{Poisson}(E_k R_k) \quad \text{for } k = 1, \ldots, n, \qquad (3)\\ R_k &= \exp(w_k\alpha + \phi_k),\\ \mathbf{w} = (w_1, \ldots, w_n) &\sim \text{N}(\mu\mathbf{1}, \Sigma_1),\\ \boldsymbol{\phi} = (\phi_1, \ldots, \phi_n) &\sim \text{N}(\mathbf{m}, \Sigma_2). \end{aligned}$$

The expected counts E_k are generated from a uniform distribution on the range $[70, 130]$, giving a moderate disease prevalence in terms of the existing literature. The risk of disease in area k, R_k is modelled on the log scale by two components. The first is a vector of air pollution concentrations denoted by $\mathbf{w}$, which are generated from a multivariate Gaussian distribution with mean $\mu = 20$ and a variance matrix Σ_1 specified by the Matérn family of autocorrelation functions. For the latter the smoothness parameter equals 1.5, the range parameter equals 60 and the standard deviation parameter is 4.5. The corresponding regression parameter is $\alpha = 0.024$, which is similar to the estimated effect sizes presented in Section 5 of the main paper. The second component in the risk generation model is the residual (confounding) spatial autocorrelation $\boldsymbol{\phi}$, which is generated from a multivariate Gaussian distribution with mean $\mathbf{m}$ and variance Σ_2, the latter again being specified by the Matérn family of autocorrelation functions. The spatial range (controlling the minimum

distance at which pairs of areas are uncorrelated) for Σ_2 is varied in the simulation study between 0, 20 and 60, to investigate its impact on model performance. In all cases the spatial autocorrelation between a pair of areas depends on the distance between their centroids (central points).

The mean function $\mathbf{m}$ for $\boldsymbol{\phi}$ is either chosen to be the constant vector of zeros, or a vector containing three distinct values, $\{-1, 0, 1\}$. Specifying a constant vector of zeros results in globally smooth residual spatial autocorrelation (used in scenarios A-C), while setting it equal to the vector with distinct values, $\{-1, 0, 1\}$ results in localised spatial autocorrelation (used in scenarios D-F). For the latter, if two neighbouring areas have the same mean value then their residuals are similar (corresponding to spatial smoothness), while if they have different mean values then their residuals are not similar (corresponding to no spatial smoothness).

The template for this localised smoothness is presented in panels D to F of Figure 1 where in each case the three distinct mean levels are evident. This figure displays example realisations from the residual spatial structures $\boldsymbol{\phi}$ generated under scenarios A to F, which differ in both the presence (A-C) or absence (D-F) of localised smoothness, and the type of global smoothness assumed. For the latter A and D correspond to independence in space, B and E are globally spatially smooth with a smaller range parameter (less smooth) than the pollution covariate, while C and F are globally spatially smooth with the same range as the pollution covariate.

[Figure 1 about here.]

5. SIMULATION STUDY - SENSITIVITY TO G

In this section we present an additional simulation study, that assesses the sensitivity of **Model-Local** to the choice of the number of intercept terms G. The study design is identical to that used in the first simulation study in the main paper, and full details are given in Section 4 of the main paper and Section 4 of this supplementary material. We consider values of $G = 3, 4, 5, 7$ in this study, where in scenarios A to C the true value that generated the data is $G = 1$ (globally smooth residual spatial autocorrelation) while for scenarios D to F the true value is $G = 3$. We thus compare $G = 5$ used in the main paper against alternative odd values of G ranging between 3 and 7, with odd values being chosen because it allows the prior to shrink the group indicators $\mathbf{Z} = (Z_1, \ldots, Z_n)$ towards a single group G^*. To assess the performance of the model when G is even, and hence when the prior shrinks $\mathbf{Z}$ equally towards two groups, we also consider $G = 4$ here.

The results of this study are presented in Table 1 in this supplementary material, which has the same format as Table 2 in the main paper. The table shows little sensitivity to the choice of G across the range of 12 scenarios used in this study. All values of G result in negligible bias, with percentage biases being less than 1.1% in all cases. The RMSE values are also very similar across the different values of G, with little systematic differences for any of the scenarios. The only slight differences are observed in scenarios D-F with $\text{SD}_{\boldsymbol{\phi}}$=0.1, where the RMSE is slightly lower for the true value of $G = 3$ compared with the other values. Finally, the coverages are also very similar across the different values of G, with the only notable differences being for scenarios D-F with $\text{SD}_{\boldsymbol{\phi}}$=0.1, where the coverages show small decreases as G increases away from its true value of 3.

[Table 1 about here.]

REFERENCES

Hughes J, Haran M (2013) Dimension reduction and alleviation of confounding for spatial generalized linear mixed models. Journal of the Royal Statistical Society Series B 75:139–159

Lee D, Mitchell R (2013) Locally adaptive spatial smoothing using conditional autoregressive models. Journal of the Royal Statistical Society Stries C 62:593–608

Rue H, Martino S, Chopin N (2009) Approximate bayesian inference for latent gaussian models using integrated nested laplace approximations (with discussion). Journal of the Royal Statistical Society Series B 71:319–392

FIGURES

Figure 1. Maps displaying example residual spatial autocorrelation structures generated in the simulated data for scenarios A to F in the first simulation study in the main paper.

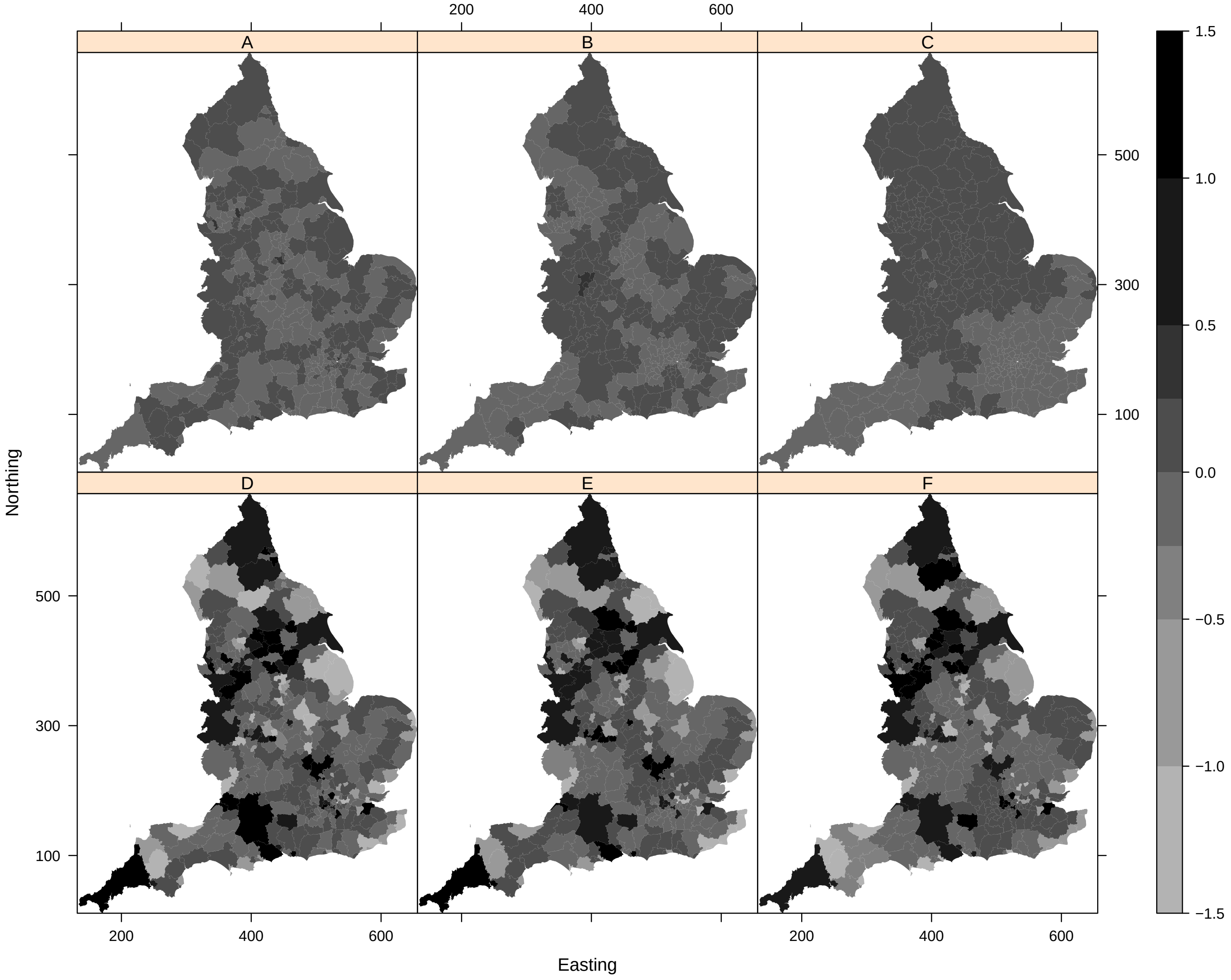

TABLES

Table 1. Results of a sensitivity analysis for **Model-Local** to different values of G. The top panel displays the bias (as a % of the true value) for the pollution-health relationship, the middle panel displays the root mean square error (as a % of the true value), while the bottom panel displays the coverage probabilities (as a %) of the 95% uncertainty intervals.

**Scenario**	SD$_{\phi}$	**Value of G**			
		$G = 3$	$G = 4$	$G = 5$	$G = 7$
**Bias**					
A	0.1	-0.21	-0.12	-0.34	-0.34
	0.01	0.06	0.05	0.07	0.06
B	0.1	-0.79	-0.80	-0.80	-0.79
	0.01	-0.19	-0.20	-0.18	-0.19
C	0.1	-0.86	0.92	0.95	0.91
	0.01	0.10	0.10	0.10	0.09
D	0.1	-0.30	0.23	-0.11	-0.40
	0.01	-0.21	-0.14	-0.11	-0.28
E	0.1	0.39	1.09	0.81	0.30
	0.01	0.30	0.33	0.31	0.28
F	0.1	0.38	0.72	0.93	0.77
	0.01	-0.01	0.03	-0.04	-0.09
**RMSE**					
A	0.1	6.41	6.37	6.45	6.45
	0.01	4.46	4.46	4.46	4.45
B	0.1	15.43	15.51	15.28	15.48
	0.01	5.00	4.99	4.91	5.00
C	0.1	19.22	19.90	18.91	19.17
	0.01	5.17	5.17	4.74	5.17
D	0.1	7.34	8.00	7.60	7.86
	0.01	5.27	5.53	4.84	5.36
E	0.1	16.45	17.50	16.88	16.96
	0.01	4.64	4.71	4.70	4.68
F	0.1	21.55	22.35	22.64	23.37
	0.01	4.62	4.61	4.64	5.13
**Coverage**					
A	0.1	95.4	95.4	94.8	96.0
	0.01	97.8	97.6	96.2	97.6
B	0.1	84.4	81.6	85.6	83.8
	0.01	95.4	94.8	95.6	95.4
C	0.1	73.6	70.0	77.0	73.2
	0.01	95.0	94.4	96.2	94.8
D	0.1	95.4	92.8	94.4	91.0
	0.01	95.8	94.4	94.6	95.2
E	0.1	79.6	77.6	76.2	74.0
	0.01	94.6	95.0	94.0	95.0
F	0.1	67.2	63.6	63.2	58.8
	0.01	95.8	95.6	95.6	95.6
